# New Perkinsea Parasitoids of Dinoflagellates Distantly Related to Parviluciferaceae Members

**DOI:** 10.3389/fmicb.2021.701196

**Published:** 2021-08-05

**Authors:** Albert Reñé, Elisabet Alacid, Rachele Gallisai, Aurélie Chambouvet, Alan D. Fernández-Valero, Esther Garcés

**Affiliations:** ^1^Departament de Biologia Marina i Oceanografia, Institut de Ciències del Mar (CSIC), Barcelona, Spain; ^2^Living Systems Institute, School of Biosciences, College of Life and Environmental Sciences, University of Exeter, Exeter, United Kingdom; ^3^Department of Zoology, University of Oxford, Oxford, United Kingdom; ^4^CNRS, Univ Brest, IRD, Ifremer, LEMAR, Plouzané, France

**Keywords:** parasitoids, protist, HAB, *Parvilucifera*, phylogeny, evolution

## Abstract

Perkinsea is a phylogenetic group of protists that includes parasites of distantly related hosts. However, its diversity is still mainly composed of environmental sequences, mostly obtained from freshwater environments. Efforts to isolate and culture parasitoids of dinoflagellates have led to the description of several phylogenetically closely related species constituting the Parviluciferaceae family. In this study, two new parasitoid species infecting dinoflagellates during recurrent coastal blooms are reported. Using the ribosomal RNA (rRNA) gene phylogenies, we show that both cluster within Perkinsea, one of them at the base of Parviluciferaceae and the other in a distinct branch unrelated to other described species. The establishment of host-parasite lab cultures of the latter allowed its morphological characterization, resulting in the formal description of *Maranthos nigrum* gen. nov., sp. nov. The life-cycle development of the two parasitoids is generally the same as that of other members of the Parviluciferaceae family but they differ in the features of the trophont and sporont stages, including the arrangement of zoospores during the mature sporangium stage and the lack of specialized structures that release the zoospores into the environment. Laboratory cross-infection experiments showed that the parasitoid host range is restricted to dinoflagellates, although it extends across several different genera. The maximum prevalence reached in the tested host populations was lower than in other Parviluciferaceae members. The findings from this study suggest that Perkinsea representatives infecting dinoflagellates are more widespread than previously thought.

## Introduction

The class Perkinsea was erected to encompass parasitic species of the genus *Perkinsus*, being characterized by having an incomplete conoid (Levine, 1978). Perkinsea was initially classified within the Apicomplexa phylum, comprising parasitic organisms with an apical complex consisting of a conoid, rhoptries, micronemes, and microtubules. Ultrastructural studies on *Perkinsus* representatives questioned their inclusion within Apicomplexans (Perkins, 1988). Early phylogenetic analyses of ribosomal genes allowed to determine their relationship with Apicomplexa (Goggin and Barker, 1993), and evidenced they were closer to Dinoflagellata (Siddall et al., 1997). The description of *Parvilucifera infectans*, by Norén et al. (1999), noted the close phylogenetic relationship of this species with those of *Perkinsus*. Like *Perkinsus*, *Parvilucifera* species have an incomplete conoid, and the formation by the two genera of a clade independent of Apicomplexa led to the establishment of the phylum Perkinsozoa (Norén et al., 1999). Within this phylum, the class Perkinsea currently includes the family Perkinsidae, which encompasses *Perkinsus* species that infect bivalves (Andrews, 1988), the family Parviluciferaceae, comprising genera of closely related parasitoids (parasites that ultimately kill their hosts) of dinoflagellates (Reñé et al., 2017a), and the family Xcellidae whose member species infect fish (Freeman et al., 2017). Recently, sequences of the agent of severe Perkinsea infection (SPI), responsible for tadpole mortality events in the United States (Isidoro-Ayza et al., 2017, 2019), were shown to cluster with environmental sequences and to form a highly supported clade referred to as Novel Alveolate Group 01 (“NAG01”), following Chambouvet et al. (2015). Consequently, the Perkinsea class is exclusively composed of parasitic species. However, besides the above-noted species and groups, Perkinsea contains a vast diversity and is mostly represented by environmental sequences with unknown correspondence.

Perkinsea are present in diverse aquatic environments, including freshwater, marine, and deep-sea sediments (Bråte et al., 2010; Lepère et al., 2010; Mangot et al., 2011), but most of its environmental sequences have been obtained in freshwater systems. Those sequences cluster into numerous independent clades or subgroups whose phylogenetic relationships are independent of their origin, since some clusters include both marine and freshwater sequences (Bråte et al., 2010; Chambouvet et al., 2014; Jobard et al., 2020). The marine families Xcellidae and Perkinsidae show a close phylogenetic relationship (Jobard et al., 2020) whereas the SPI agent clusters with environmental sequences deriving only from freshwater environments. Parviluciferaceae is a distinct branch representing various marine environments and encompassing parasitoid species that infect a wide range of dinoflagellates (Reñé et al., 2017a). To date, it includes six species of the genus *Parvilucifera* (Norén et al., 1999; Figueroa et al., 2008; Lepelletier et al., 2014; Reñé et al., 2017b; Alacid et al., 2020; Jeon and Park, 2020) and species of the genera *Snorkelia*, *Dinovorax*, and *Tuberlatum* (Leander and Hoppenrath, 2008; Reñé et al., 2017a; Jeon and Park, 2019). One of the main morphological features used to distinguish among the different genera of Parviluciferaceae is the structure used by these organisms to release zoospores into the environment: small apertures covered by opercula in *Parvilucifera*, short tubes in *Tuberlatum*, and long tubes in *Snorkelia* and *Dinovorax*.

All of the species belonging to those genera have been detected and isolated from coastal areas where dinoflagellate blooms are common. The high cellular abundances of predominant species within the phytoplankton community promote the propagation of parasitoid infections (Alacid et al., 2017; Reñé et al., 2021). In this study, two parasitic species infecting the dinoflagellates *Barrufeta bravensis* and *Alexandrium taylorii* were isolated during blooms occurring at beaches along the Catalan coast (NW Mediterranean Sea). Single-cell isolation and 18S rRNA gene amplification enabled a determination of the phylogenetic position of one parasitic species, hereafter named Perkinsea ex *Barrufeta bravensis*. The second species was successfully cultured and detailed information on its morphological, molecular, and ultrastructural features and on its life-cycle stages was obtained. Accordingly, the species *Maranthos nigrum* gen. nov. et sp. nov. is proposed. Using single-sporangium amplification of the rRNA gene obtained from an established culture, we demonstrate that the latter species is the first representative infecting dinoflagellates forming an undescribed distinct branch within Perkinsea.

## Materials and Methods

### Isolation and Culture

Seawater was collected along the shore of four beaches of the Catalan coast (NW Mediterranean Sea) during summer months between 2015 and 2018 (Table 1). In the summer of 2015, a monospecific bloom of the dinoflagellate *Barrufeta bravensis* was detected at Nova Icària beach (Barcelona), with a density of 6.7 × 10^6^ cells⋅L^–1^. In 2016, 2017, and 2018, high abundances (>10^5^ cells⋅L^–1^; >75% of dinoflagellate cells) of *Alexandrium taylorii* developed at different beaches. Seawater samples of 1–2 L were concentrated to a volume of 50 mL using a 10-μm net mesh, and subsamples of ∼5 mL were transferred to 6-well plates. The plates were incubated in a culture chamber (temperature 20°C, 10:14 light:dark cycle, irradiance of 100 μmol photons m^–2^ s^–1^). After several days, infections of *B. bravensis* and *A. taylorii* cells by parasitoids were observed. The infected cells were manually isolated and transferred to 24-well plates. Because *B. bravensis* cultures were not available in the culture collection of Institut de Ciències del Mar (ICM-CSIC), other dinoflagellate species were added to the samples as possible hosts, but the *B. bravensis* parasitoid could not be propagated in culture. Instead, infections in the field sample allowed observations of the parasitoid’s life-cycle and yielded molecular sequences. Cultures established for the parasitoid of *A. taylorii* (Table 1) were maintained by transferring a portion of the infected cells twice weekly into new wells containing a healthy host culture. Unfortunately, the host culture of *A. taylorii* was lost, such that the ability of the parasite to infect other hosts was tested, by adding other species belonging to the *Alexandrium* genus available in our culture collection. Successful infection of a mixture of *A. minutum* and *A. mediterraneum* allowed the maintenance and propagation of the parasitoid.

**TABLE 1:** Information on the detections of parasitoids and molecular sequences obtained.

Species	Strain	Host	Abundance	Location	Coordinates	Date	Cultured	SSU rRNA	LSU rRNA
Perkinsea *ex Barrufeta bravensis*	Nova Icària 2015	*B. bravensis*	6.7 × 10^6^ cell/L	Barcelona (Catalonia)	41° 23′ 24″ N; 2° 12′ 11″ E	Jul 2015	No	MT649884	
*Maranthos nigrum*	Fosca 2016	*A. taylorii*	5.4 × 10^5^ cell/L	La Fosca (Catalonia)	41° 51′ 27″ N; 3° 8′ 38″ E	Jul 2016	Yes	MN721813	
*Maranthos nigrum*	Estartit 2016	*A. taylorii*	5.0 × 10^5^ cell/L	L’Estartit (Catalonia)	42° 3′ 5″ N; 3° 12′ 9″E	Jul 2016	Yes		MN721817
									MN721818
									MN721819
*Maranthos nigrum*	Estartit 2017	*A. taylorii*		L’Estartit (Catalonia)	42° 3′ 5″ N; 3° 12′ 9″ E	Jul 2017	Yes	MN721814	
*Maranthos nigrum*	Sant Pol 2018	*A. taylorii*	3.0 × 10^6^ cell/L	Sant Feliu de Guíxols (Catalonia)	41° 47′ 26″ N; 3° 3′ 5.4″ E	Jul 2018	Yes	MN721815	

### Single-Sporangia PCR Amplification of the SSU and LSU rRNA Regions and Sequencing

Three to five mature sporangia were isolated from samples of the *B. bravensis* parasitoid and from the established cultures of the novel parasitoid *M. nigrum* using a capillary micropipette. The samples were transferred to successive drops of seawater, placed in 200-μL PCR tubes, subjected to several rounds of freeze-thaw, and finally stored at –80°C until processed.

The SSU rRNA gene from single sporangia of strain Estartit 2016 and from the Nova Icària beach isolates was amplified in 50-μL reactions containing 5 μL of 10 × buffer (with 1.25 mM MgCl_2_), 200 μM each dNTP, 0.5 mM of primers EUK A and 1209R, and 2.5 U of Taq DNA polymerase (Invitrogen). The PCR conditions were as follows: initial denaturation for 3 min at 94°C, 40 cycles of 45 s at 94°C, 1 min at 55°C, and 3 min at 72°C, followed by a final extension step for 10 min at 72°C. The LSU rRNA gene was amplified using the primer pair D1R– D3B (Scholin and Anderson, 1994; Nunn et al., 1996) and the above-described PCR mixture under the following PCR conditions: initial denaturation for 5 min at 95°C, 40 cycles of 20 s at 95°C, 30 s at 55°C, and 1 min at 72°C, followed by a final extension step for 7 min at 72°C.

The SSU rRNA genes of strains Estartit 2017 and Sant Pol 2018 were amplified in several steps. First, a PCR was conducted using the primers EK-82F–28S-1611R (López-García et al., 2001; Moreira et al., 2007), 2 μL of 10 × buffer (TaKaRa Bio), 1.5 mM MgCl_2_, 1 U of TaKaRa Taq DNA polymerase (TaKaRa Bio), 0.2 mM of each dNTP, and 0.4 mM of each primer. The PCR conditions were as follows: initial denaturation for 3 min at 95°C, followed by 6 cycles of 15 s at 95°C, 30 s at 58–53°C, decreasing 1°C each cycle, 2 min at 72°C, and 34 additional cycles at an annealing temperature of 52°C, followed by a final extension step for 5 min at 72°C. Second, a semi-nested PCR was performed using 1 μL of previous PCR product as template and the primer pair EK-82F–EK-1520R (López-García et al., 2001). The PCR mixture contained 1 μL of template, 2.5 μL of 10 × buffer (with 1.5 mM MgCl_2_), 1.25 U of Platinum Taq DNA polymerase (Invitrogen, Thermo Fisher Scientific Corp.), 0.2 mM of each dNTP, and 0.4 mM of each primer. The PCR conditions were as follows: initial denaturation for 2 min at 94°C, 35 cycles of 15 s at 94°C, 30 s at 52°C, 1 min at 72°C, and a final extension step for 5 min at 72°C. A second semi-nested PCR was performed using 1 μL of the previous PCR product as template, the primer pair EK-82F–1209R (Giovannoni et al., 1988), and the same PCR conditions as above. All PCR products were visualized under 1.2% agarose gel electrophoresis, followed by their purification and external sequencing at Genoscreen (France) on a 3730XL DNA sequencer. PCR samples were sequenced double-stranded using the previously described forward and reverse primers. The final sets of sequences were assembled into consensus sequences using Geneious v.10. All sequences obtained were deposited in GenBank under the accession numbers MN721813-MN721815 and MT649884 for SSU rRNA and MN721817-MN721819 for LSU rRNA sequences (Table 1).

### Multiple Sequence Alignment and Phylogenetic Analysis

The SSU rRNA sequences were assembled for multiple sequence alignment with 83 published sequences recovered from the NCBI nr database and representing the main molecular diversity of Perkinsea. SSU rRNA sequences belonging to four dinoflagellates (accession numbers: M88521, U27500, AF080096, AF022200), two belonging to Syndiniales group II (EF065717, AF472555), and one belonging to Syndiniales group I (AB264776) served as the outgroup. The sequences were aligned using the MAFFT v7.2 iterative refinement method Q-INS-i (Katoh and Standley, 2013). The alignment was manually masked using Seaview v5.0.4 (Gouy et al., 2021), resulting in an alignment of 1,434 positions. The maximum likelihood phylogenetic tree was reconstructed with IQ-TREE v1.6.11^1^ under the GTR + F + R9 model, determined based on a best fitting of the data by ModelFinder, as implemented in IQ-TREE, and on the Akaike information criterion (AIC) with 10,000 ultrafast bootstrap replicates. Non-parametric bootstraps (1,000 pseudoreplicates) were reconstructed using RAxML HPC2 on XSEDE v8.2.12, with CATGTR as the model (available on http://www.phylo.org/) to evaluate node supports. In addition, Bayesian posterior probabilities were calculated using parameters from the software MrBayes v3.2.6 (available on http://www.phylo.org/), model GTR + Γ (lset, nst = 6 rates = gamma), based on the AIC; the covarion parameter was then selected. The chains were run for 2,000,000 generations with two replicate tree searches, both with four MCMC chains with a heat parameter of 2. Trees were sampled every 250 generations. In both analyses, the MCMC searches converged within the first 25% of the generations sampled, such that the first 1/4 of the search results were discarded (as the burn-in). The consensus topologies and posterior probabilities of each node were then calculated from the remaining trees.

### Infection Dynamics

The development of infections of *B. bravensis* was determined directly, by observing field samples obtained from Nova Icària beach. Early infections of dinoflagellate cells by the cultured parasitoid strains Estartit 2016 and Sant Pol 2018 were transferred to 2-mL settling chambers and the morphology and development of the different life-cycle stages of the parasitoids were studied and documented. All observations were done under a phase-contrast Leica DM-IRB inverted microscope (Leica Microsystems, Wetzlar, Germany) connected to a ProgRes C10 digital camera (JENOPTIK Laser, Optik, Systeme GmbH, Jena, Germany).

Samples from strains Estartit 2016 and Sant Pol 2018 were processed for scanning electron microscopy (SEM) to characterize the morphology of the zoospores and other life-cycle stages of the parasitoid. When high numbers of motile zoospores were present, 2 mL of the culture was subsampled and filtered through a 5-μm filter to remove host cells and other life-cycle stages. The filtrate, containing the zoospores, was collected in a 2-mL Eppendorf tube. Another 2-mL subsample was collected when mature sporangia were observed in the culture. The subsamples were fixed with 2% glutaraldehyde (final concentration) and stored at 4°C until processed. The samples were gravity-filtered through polycarbonate filters of 0.8-μm (zoospores) and 10-μm (sporangia) pore size, washed for 15 min in autoclaved seawater followed by 15 min in distilled water, and then dehydrated in an ethanol series (25, 50, 75, 90, 96, and 100; ca. 10 min/step). The final step of 100% ethanol was repeated twice. The filters were critical-point dried, mounted on stubs, sputter-coated with gold-palladium, and examined under a JEOL JSM-6500F scanning electron microscope (JEOL-USA Inc., Peabody, MA, United States) at the Servei de Microscopia Electrònica (ICM-CSIC).

The ultrastructure of the different life-cycle stages was examined under transmission electron microscopy (TEM). The sample consisted of 2 mL of culture collected on three successive days during the infection process and then fixed with 2% glutaraldehyde. Sample processing and TEM were performed at the CCiTUB (Barcelona) following the procedures detailed in Reñé et al. (2017b).

### Host-Range Screening and Prevalence Curve

Forty-four different strains of phytoplankton culture, representing 42 species, were acquired from the culture collection of the Centro Oceanográfico de Vigo (CCVIEO), Spain, and the culture collection of the Institut de Ciències del Mar (ICM-CSIC), Barcelona, Spain. The cultures covered several phytoplankton phyla: dinoflagellates (31 species, including representatives of five orders), diatoms (3 species), chlorophytes (3 species), haptophytes (4 species), raphidophytes (2 species), and cryptophytes (1 species) (Supplementary Table 1). The cultures were maintained in 50-mL polystyrene tissue culture flasks containing silica-free L1 medium (Guillard and Hargraves, 1993), except in the case of diatoms, which were cultured in L1 + Si medium. All phytoplankton cultures were maintained at 21°C (except *Dinophysis acuminata* and *Alexandrium catenella*, maintained at 16°C) in culture chambers with a L:D cycle of 10:14 h and irradiation at 100 μmol photons m^2^ s^–1^.

The susceptibility of the phytoplankton strains to infection by *M. nigrum* was tested in 24-well plates in a final volume of 2 mL and a host cell:zoospore ratio of 1:10. All procedures were performed for each of the two parasitoid strains, Estartit 2017 and Sant Pol 2018. One mL of phytoplankton culture was fixed in Lugol’s iodine solution (2%) and counted using a Sedgewick-Rafter chamber. A living subsample was then added to each well at a final concentration between 10^4^ and 10^3^ cells mL^–1^. When a high number of free-living zoospores was observed in the parasitoid culture, 3–5 mL of the culture was filtered through a 3-μm polycarbonate filter in order to separate the zoospores from the host cells and from other life-cycle stages of the parasitoid. The flow-through fraction was collected and a 1-mL subsample was fixed with formaldehyde solution at a final concentration of 4%. The zoospores were counted as previously described and then added to each well at a host cell:zoospore ratio of 1:10 in all host-range experiments.

The phytoplankton-parasitoid co-cultures were examined daily by inverted light microscopy to detect the presence of infections of the phytoplankton strains. When infections were detected, their viability was checked by adding healthy host cells 5 days after the initial inoculation, to allow a second round of infection. If infections were not detected after 5 days from the initial parasitoid inoculation, recently produced zoospores were added to the same well in order to confirm host resistance to the parasitoid. The co-cultures were observed for 15 days in both cases. The susceptibility of the different hosts was determined qualitatively for both strains following Lepelletier et al. (2014), resulting in their classification as resistant (no infection observed), moderately resistant (the host is infected but >10 host cells remain after 15 days), moderately sensitive (the host is infected and <10 host cells survive the infection), or sensitive (no host survives the infection). The prevalence curve was assessed on the *A. minutum* host (Arenys strain), based on its high infection level among the tested dinoflagellate species. Zoospores were separated by filtering 20 mL of parasitoid culture through a 10-μm polycarbonate filter into a 50-mL tube. One mL of the filtered sample and the *A. minutum* culture were fixed in 10% formalin (v:v) and cell abundances were determined under light microscopy using a Sedgewick-Rafter counting chamber. Subsequently, both cultures were mixed in 6-well culture plates at volumes resulting in host cell:zoospore ratios of 1:5, 1:10, 1:20, 1:40, 1:80, with three replicates per ratio. All plates were incubated for 48 h at 21°C in culture chambers with a L:D cycle of 10:14 h at 100 μmol photons m^2^ s^–1^. Finally, 1 mL of each sample was preserved with 10% (v:v) formalin and the proportion of infected host cells was determined under light microscopy using a Sedgewick-Rafter counting chamber.

## Results

### Phylogenetic Relationships

**FIGURE 1:**
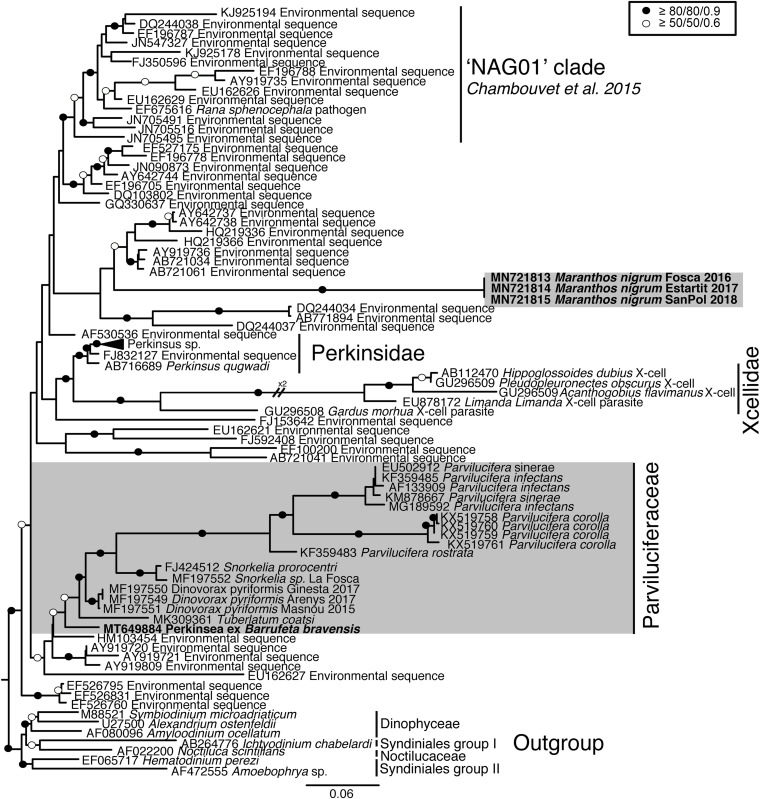
Maximum likelihood phylogenetic tree inferred in IQ-TREE with GTR + F + R9 model from the SSU rRNA gene sequences of Perkinsea. Sequences of Dinophyceae and Syndiniales served as the outgroup. Value at branches correspond to ultrafast bootstrap (10,000 replicates, GTR + F + R9 model), non-parametric bootstrap (1000 replicates, CATGTR model) and Bayesian posterior probabilities (2,000,000 generations, GTR + G model). Support values are summarized by black circles when all are ≥80%/80%/0.9, and a white circle when topology support is weaker but all values are ≥50%/50%/0.6. Sequences obtained in this study are indicated in bold; the shaded area indicates parasitoids of dinoflagellates.

SSU rRNA gene sequences of the three cultured strains corresponding to *M. nigrum* (length = 769–1094 bp) had an identity of 100%, with only 1–3 differences, all corresponding to ambiguous (Ns) positions. The highest similarity to the sequences available in GenBank (85.9%) was that of environmental sequence AY919720. The closest match of the SSU rRNA gene sequence assigned to Perkinsea ex *Barrufeta bravensis* (length = 1350 bp) was with sequences belonging to *Dinovorax pyriformis* (93.5–94.7% similarity). According to the SSU rRNA gene phylogeny (Figure 1), the Perkinsea sequences formed a monophyletic group (100% ultrafast bootstrap/94% non-parametric bootstrap/1 Bayesian posterior probability) grouped in three main clades. The first (74%/–/–) included many environmental sequences and the “NAG01” clade (99%/86%/1), encompassing the sequence of the “SPI” agent. The sequences of *M. nigrum* clustered within this first main clade but formed a distinct long branch completely independent of the other known Perkinsea species and not statistically supported by the closest environmental sequences. The second main clade (81%/41%/0.94) comprised one clade with all Perkinsidae representatives, corresponding to *Perkinsus* species (99%/88%/1), and another clade with maximum statistical support and containing Xcellidae species. The third main clade included all sequences belonging to Parviluciferaceae; these clustered together with moderate-low support (85%/58%/0.89). The sequence of Perkinsea ex *Barrufeta bravensis* clustered at the base of the Parviluciferaceae clade. All *Parvilucifera* species formed a clade with maximum support. *Snorkelia* sequences formed a sister clade to *Parvilucifera* species (87%/76%/0.9), followed by *Dinovorax pyriformis* (95%/69%/0.88). *Tuberlatum coatsi* clustered at the base of the previously known Parviluciferaceae species (91%/69%/0.89).

As the LSU rRNA gene sequences of Perkinsea are limited to *Perkinsus* representatives and Parviluciferaceae members, there is little phylogenetic information. In the phylogenetic tree (Supplementary Figure 1), Perkinsea members clustered together, forming a highly supported clade (97% bootstrap/1 Bayesian posterior probability). All Perkinsidae sequences formed a clade with maximum statistical support, and the Parviluciferaceae sequences clustered together. Within this clade, all *Parvilucifera* sequences formed a cluster with maximum statistical support. Sequences of *D. pyriformis* and *T. coatsi* also clustered together (82%/1), forming a sister clade to *Parvilucifera*, albeit with low statistical support. The sequences corresponding to *M. nigrum*, determined to be identical, clustered at the base of Parviluciferaceae and formed a highly supported cluster with the respective sequences (96%/1).

### Morphology of the Life-Cycle Stages and Their Development

Perkinsea *ex Barrufeta bravensis* was detected during a *B. bravens*is bloom. After infecting the host cells, it developed a trophont (Supplementary Figures 2A,B). Occasionally, double infections of the same cell occurred and resulted in two growing trophonts (Supplementary Figure 2C). Once the host was consumed, the parasitoid formed an early sporont, followed by the gradual development of new zoospores (Supplementary Figure 2D). When the sporangium had matured, it became filled with zoospores and acquired a pale coloration (Supplementary Figure 2E). Eventually, the zoospores were released into the environment through several apertures in the sporangium wall (Supplementary Figure 2F). The sigmoid shaped free-living zoospores (not shown) then infected new host cells.

**FIGURE 2:**
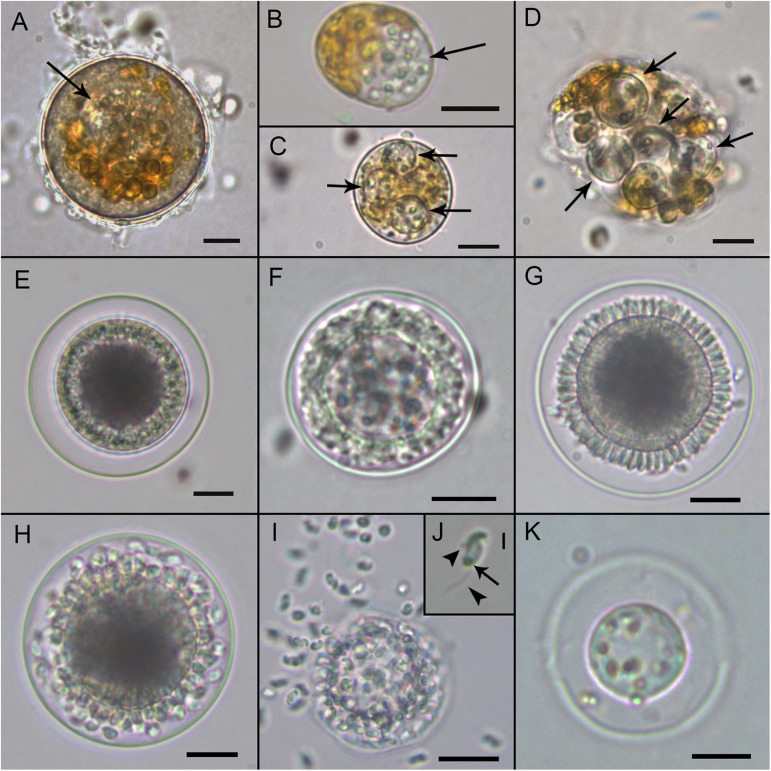
Light microscopy micrographs showing the life-cycle stages of *Maranthos nigrum* (strain Sant Pol 2018) during an infection of *Alexandrium taylorii.* **(A)** Early infection on an *A. taylorii* cell. **(B)** Trophont (arrow) developing inside an elongated/ecdysed host cell. **(C)** Three hyaline trophonts (arrows) containing many refractive small bodies. **(D)** Multiple late trophonts (arrows) on a single host cell, showing cytoplasm accumulation in the periphery. **(E)** An early sporont begins to accumulate organelles in the cytoplasm. **(F)** Sporont showing the early formation of zoospores in the periphery of the inner membrane. **(G)** Late sporont showing the budding process of zoospore formation arranged around a central blackish body. **(H)** Mature sporangium with zoospores detaching from the central body. **(I)** Zoospores emerging from the sporangium after the rupture of the external wall. **(J)** The typical sigmoid shape of the zoospores. Arrowheads indicate anterior and posterior flagellum, and the arrow indicates the refractive body. **(K)** Empty sporangium with the residual body. All scale bars = 10 μm, except **(J)** = 2 μm.

**FIGURE 3:**
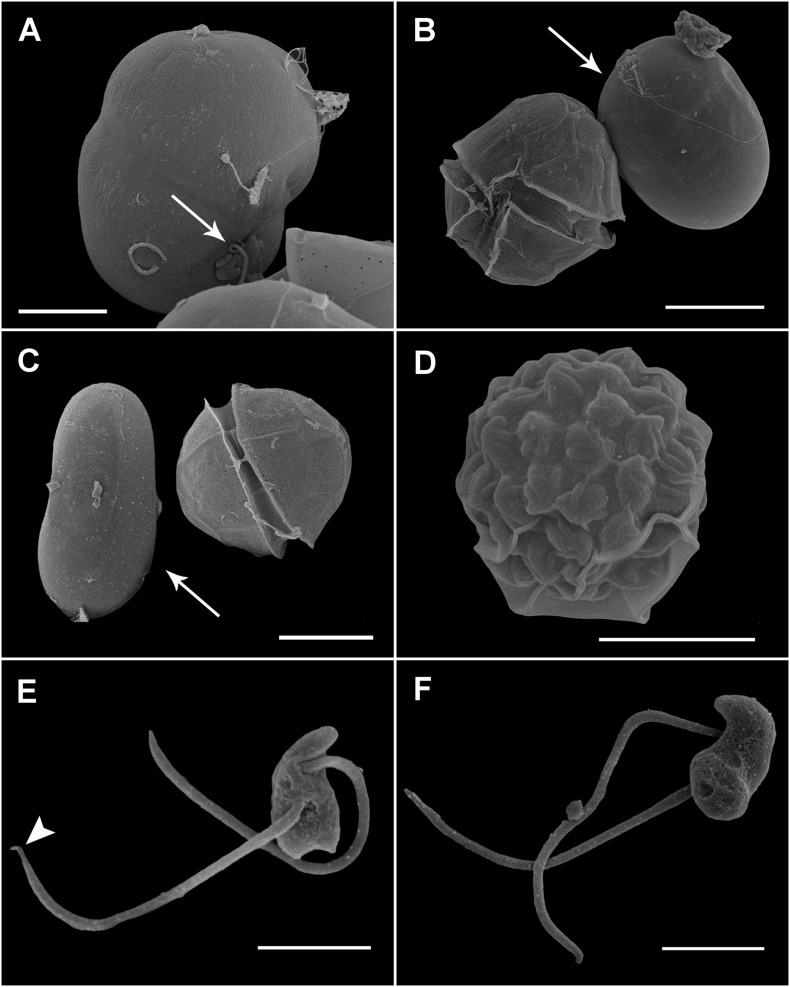
Scanning electron microscopy (SEM) of the sporangia and zoospores of *Maranthos nigrum* during infection of *Alexandrium minutum*. **(A)** Zoospore (arrow) attached to the ventral area of an *A. minutum* dinoflagellate cell that has lost its theca. **(B,C)** Infected cell during trophont development (arrow), showing an elongated shape and smooth surface. **(D)** A mature sporangium with a collapsed outer membrane reveals the outline of the zoospores. **(E)** Ventral view of a zoospore showing the insertion of two flagella of similar length. The distal shrunken posterior flagellum is seen (arrowhead). **(F)** Dorsal view of a zoospore showing its sigmoid shape. Scale bars: **(A,D)** = 5 μm, **(B,C)** = 10 μm, and **(E,F)** = 2 μm.

*Maranthos nigrum* was detected at several beaches (Table 1) during blooms of *A. taylorii*, which reached cell abundances >10^5^ cells L^–1^ and represented >75% of the dinoflagellate community. Although the gymnodinioid species *Gymnodinium litoralis* and *Levanderina fissa* were also detected, only *A. taylorii* cells became infected with the parasitoid. Infection began by the penetration of a healthy dinoflagellate host cell by a free-living zoospore (Figure 2A), usually around the host’s periflagellar ventral area (Figure 3A, arrow). The host then stopped swimming and its cytoplasmic content became highly granulated (Figure 2A). Transformation of the infective stage into a spherical trophont was accompanied by a gradual degradation of the host cytoplasm (Figure 2B). Both single (Figure 2B) and multiple, simultaneous (Figure 2C) infections were observed, with more than five infections per host cell commonly detected (Figure 2D). The trophont was hyaline and contained numerous small bodies (Figures 2B–D). During the early stage of infection, *A. mediterraneum* and *A. minutum* host cells sometimes shed their theca, via a process called ecdysis. In this case, the trophonts grew within host cells lacking a theca and therefore having an elongated shape (Figures 2B, 3B,C). The final size of the trophont depended on the size of the host and whether the host cell was infected by one or multiple parasitoids. While the trophont consumed the host cytoplasm, its own cytoplasm became visible in the periphery of the cell (Figure 2D). With complete consumption of the host, the trophont developed into a sporont, during which time the external wall detached from the internal membrane (Figure 2E). The center of the cell became occupied by a vacuole containing dark granulated material, and the peripheral cytoplasmic content became thicker and denser (Figure 2E). The zoospores then began to differentiate (schizogony) in the periphery of the cytoplasmic body (Figure 2F), gradually budding and individualizing (Figures 2G,H, 3D). At a late stage, the elongated zoospores assumed their characteristic arrangement around a large central blackish body (Figures 2G, 3D), detaching from it once they had completely matured (Figure 2H). Finally, the outer cell wall of the sporangium burst, releasing the infective zoospores into the environment without the aid of specialized release structures (Figure 2I) and leaving a large hyaline residual body inside the empty sporangium (Figure 2K). The sigmoid shaped zoospores (6 μm long and 3 μm wide; *n* = 11; Figures 2J, 3E,F) had two flagella of similar length (6–7 μm) and lacking hairs (Figures 3E,F). The anterior area of the zoospore was characterized by a rostrum (Figure 3E), with the anterior flagellum inserted ventrally below it (Figure 3E). The posterior flagellum also emerged ventrally from a median position of the zoospore body (Figure 3E) and tapered at its distal end (Figure 3E, arrowhead). Both flagella were inserted at a 90° angle (Figure 3E). Sporangia on *A. taylorii* produced zoospores at a rate of roughly 27 ± 7 (s.d.)/10^3^ μm^3^ (*n* = 2).

### Ultrastructure of the Life-Cycle Stages

**FIGURE 4:**
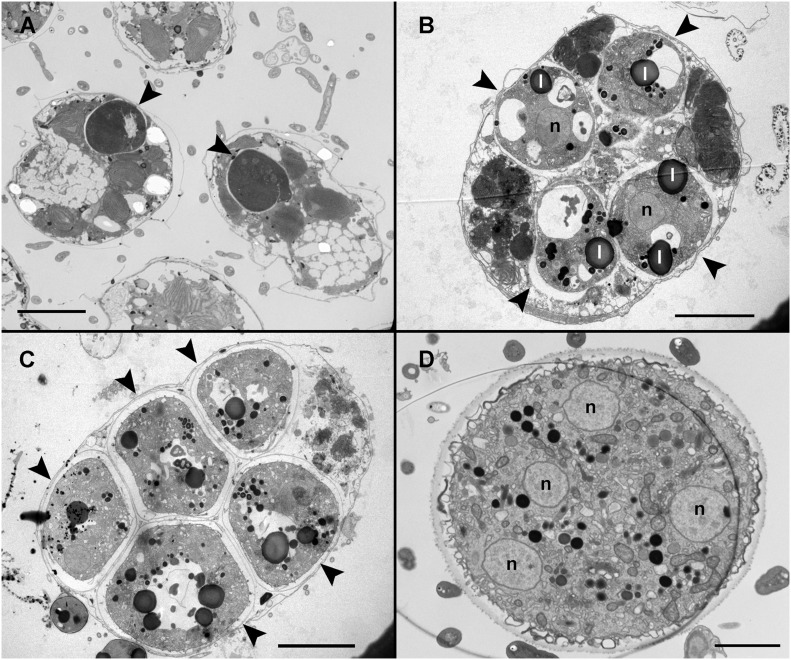
Transmission electron microscopy (TEM) of *Maranthos nigrum* showing the parasitoid’s life-cycle stages from early trophont to early sporont. **(A)** *Alexandrium minutum* cells with an early trophont of *M. nigrum* inside their host cytoplasm (arrowheads). **(B)** Four trophonts (arrowheads) developing inside a dinoflagellate cell. **(C)** Five late trophonts (arrowheads) after having completely consumed the host cytoplasm and occupying the whole dinoflagellate volume. The external membranes of the different trophonts are in close contact. **(D)** Early sporont showing several nuclei. Abbreviations: l = lipid granule; n = nucleus. Scale bars **(A)** = 10 μm, **(B,C)** = 5 μm, and **(D)** = 2 μm.

**FIGURE 5:**
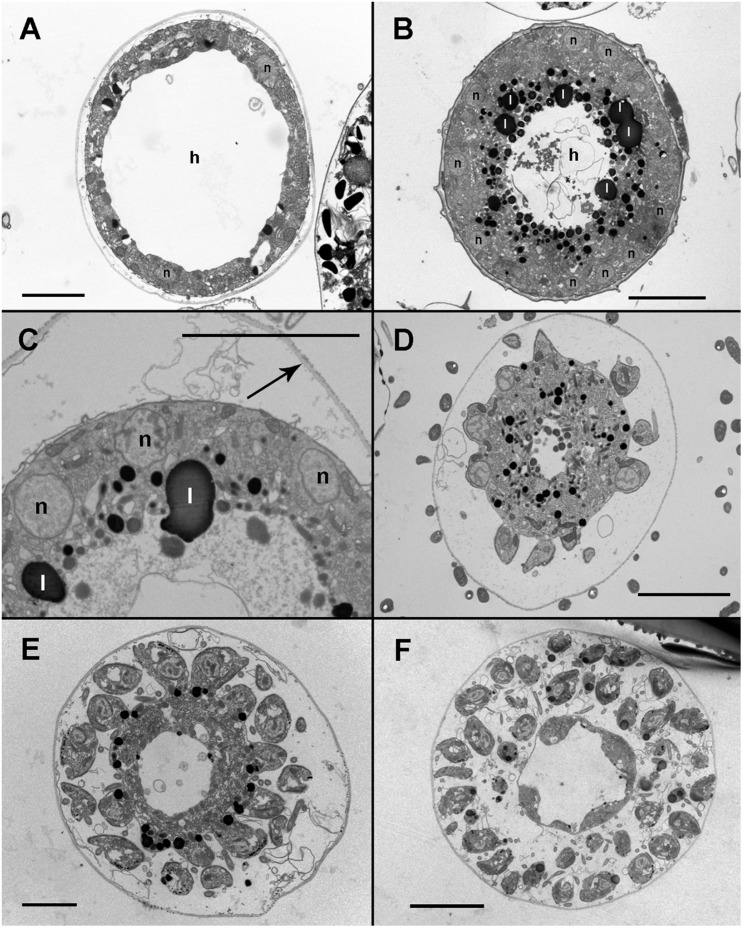
Transmission electron microscopy (TEM) micrographs of *Maranthos nigrum* showing the parasitoid’s life-cycle stages from early sporont to sporangium during infection of *Alexandrium minutum*. **(A)** Karyokinesis in an early sporont. The cytoplasm is restricted to the cell periphery and the central area is occupied by hyaline material. **(B)** A sporont with multiple nuclei. The inner cytoplasm is occupied by lipid droplets and the central area is made up of hyaline material. **(C)** Detail of the cytoplasm; several organelles are visible. Note that the external wall (arrow) is detached from the cell body. **(D)** Late sporont with zoospores beginning to form in the cell periphery. **(E)** Late sporont containing both already formed zoospores and zoospores still attached to the central body. **(F)** Sporangium showing mature zoospores detached from the central body, which has been almost completely consumed. Abbreviations: h = Hyaline material; l = lipid granule; n = nucleus. All scale bars = 5 μm, except **(A,E)** = 2 μm.

**FIGURE 6:**
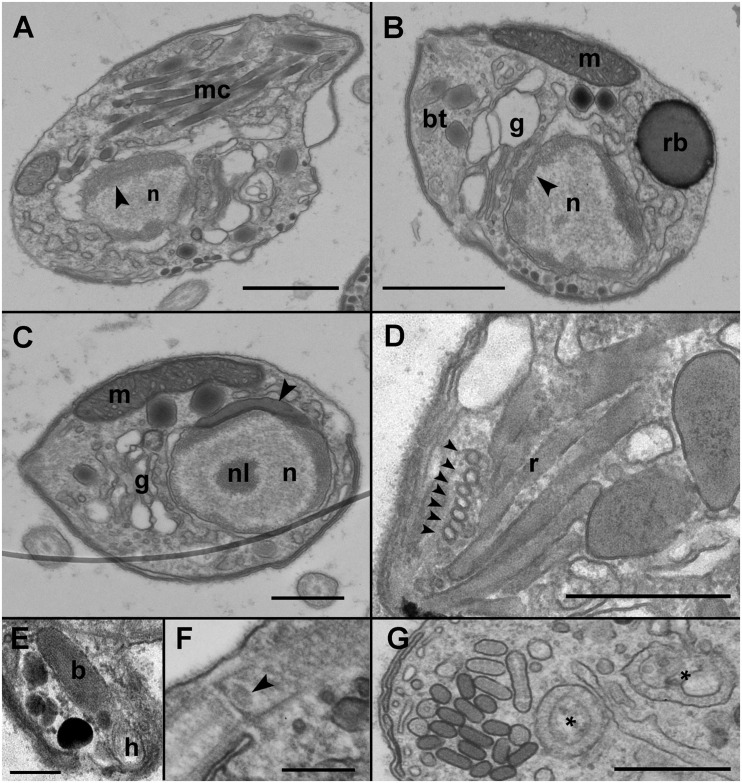
Transmission electron microscopy (TEM) micrographs showing the general features of *Maranthos nigrum* zoospores. **(A)** Longitudinal section of a zoospore showing the nucleus with heterochromatin in its periphery (arrowhead) and multiple apical complex microtubules. **(B)** Transverse section of a zoospore. The nucleus, with its heterochromatin (arrowhead), occupies a central position, and the Golgi apparatus is located close to it. The mitochondrion, trichocysts, and the refractile body are located in the periphery. **(C)** Transverse section of a zoospore showing the heterochromatin and the nucleolus inside the nucleus. An electron-dense structure is located close to the nucleus (arrowhead). The mitochondrion occupies a lateral position and the Golgi apparatus is close to the nucleus. **(D)** A section of the apical area of a zoospore shows the pseudo-conoid microtubules (arrowheads) and several rhoptries. **(E)** Detail of a bipartite trichocyst, showing the body and head. **(F)** Section of a zoospore, showing the basal body of the posterior flagellum, with a dense globule in the transition zone (arrowhead). **(G)** Detail of undefined bodies present in the zoospore cytoplasm (*). Abbreviations: b = trichocyst body; bt = bipartite trichocysts; g = Golgi apparatus; h = trichocyst head; m = mitochondrion; mc = apical complex microtubules; n = nucleus; nl = nucleolus; r = rhoptries; rb = refractile body. Scale bars: **(A,B)** = 1 μm, **(C,D,G)** = 500 nm, **(E,F)** = 200 nm.

Infected cells possessed one or multiple small spherical trophonts (Figures 4A–C, arrowheads). Early trophonts grew inside their own parasitophorous vacuole, progressively degrading the host cytoplasm (Figure 4B). Within the trophonts, a single nucleus and vacuoles filled with lipid granules were seen. Following their complete consumption of the host cytoplasm, the trophonts filled the host biovolume and no longer grew in size. In the case of multiple infections, the parasitophorous vacuoles of the trophonts were in tight contact (Figure 4C). At this stage, the nuclei began to divide, becoming multinucleated (sporont stage; Figure 4D), with the cytoplasmic content confined to the cell periphery and the central area of the cell occupied by hyaline material (Figure 5A). With the progression of karyokinesis, the number of nuclei increased, as did the number of other organelles, such as mitochondria and trichocysts. All of these structures were randomly situated inside the sporont (Figure 5B). As the cytoplasm gradually contracted, an empty space formed that divided the outer membrane from the cellular content (Figure 5C, arrow). Cytokinesis was subsequently initiated, indicated by numerous buddings at the cell periphery (Figure 5D). The zoospores began to differentiate and individualize, progressively occupying the whole volume of the sporangium, whereas the volume of the central cytoplasm became gradually smaller (Figure 5E). Finally, the completely formed zoospores detached from the central body in preparation for their release (Figure 5F). The zoospores had a single roundish nucleus with heterochromatin in the periphery (Figures 6A,B, arrowhead) and a central nucleolus (Figure 6C). An elongated mitochondrion with tubular cristae was positioned laterally (Figures 6B,C) while the Golgi apparatus was located close to the nucleus (Figures 6B,C). Bipartite trichocysts were also present in the zoospores. They were characterized by a head region made up of twisted filaments and an electron-dense body (Figure 6E), square shaped in cross section (Figure 6B). The basal body of the posterior flagellum presented a transverse septum in the transition zone and a dense globule (Figure 6F, arrowhead). The apical complex was composed of a pseudo-conoid consisting of seven microtubules (Figure 6D arrowheads), micronemes, and rhoptries (Figures 6A,D). In some zoospores, structures of unknown function but probably corresponding to basal structures of the flagellar apparatus were observed (Figure 6G, asterisk).

**FIGURE 7:**
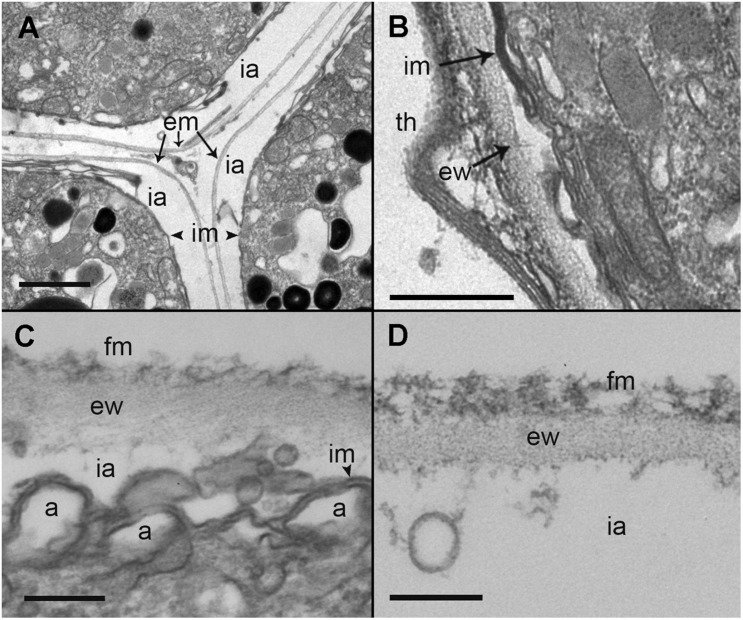
Transmission electron microscopy (TEM) micrographs showing the different stages in the development of *Maranthos nigrum* membranes. **(A)** External structure of three trophont membranes growing in contact. **(B)** Membrane structures from an early sporont. **(C)** Detail of the membrane from an early sporont, showing the folded parasitoid inner membrane with the external wall completely formed. **(D)** External wall structure after retraction of the sporont cytoplasm. Abbreviations: a = alveoli; em = external membrane; ew = external wall; fm = fibrous material; ia = intermediate area; im = inner membrane; th = dinoflagellate theca. Scale bars: **(A)** = 1 μm; **(B)** = 500 nm; **(C)**, **(D)** = 200 nm.

During the early stages of parasitoid infection, the area between the parasitophorous vacuole membrane and the plasma membrane of the trophont developed into an external membrane, with an empty intermediate area between that of the inner and external membranes (Figure 7A). At the early sporont stage, the external membrane thickened to form the external wall (Figure 7B). By the late sporont stage, a smooth external wall had formed completely and was covered by fibrous material (Figure 7C). After retraction of the cytoplasm of the sporont, during zoospore formation, the integrity of the external wall was maintained, although the empty intermediate area occupied a larger space within the sporangium (Figure 7D).

### Host-Range Characterization and Prevalence Curves

A total of 44 phytoplankton strains were tested for their susceptibility to infection by two *M. nigrum* strains. They showed very similar host ranges restricted to dinoflagellates (Supplementary Table 1). Among the 44 phytoplankton strains tested, 16 strains of dinoflagellates, representing 14 different species, could be infected by parasitoid strain Estartit 2017, and 17 strains of dinoflagellates, representing 15 different species, by parasitoid strain Sant Pol 2018. The only difference between the two parasitoid strains was the susceptibility of *Gambierdiscus excentricus*, which could be infected only by Sant Pol 2018.

Within dinoflagellates, five orders were tested. Of the 14 different strains of Gonyaulacales, 11–12 were susceptible to parasitoid infections (*G. excentricus* was infected only by Sant Pol 2018). Images of those infections can be found in Supplementary Figure 3. Among Gonyaulacales strains, 10 belonged to the *Alexandrium* genus, and all were infected except *A. margalefi*, which was resistant to infection. Other infected Gonyaulacales included *G. excentricus*, *Ostreopsis ovata*, and *Coolia tropicalis*, but not the tested *Coolia monotis* strain. All three of the tested Peridiniales strains, i.e., *Kryptoperidinium foliaceum*, *Scrippsiella trochoidea*, and *Heterocapsa triquetra*, were infected by both parasitoid strains. From the six tested Prorocentrales strains, only *Prorocentrum rathymum* and *P. lima* were infected. Finally, neither any of the seven Gymnodiniales species nor the lone Dinophysiales representative (*D. acuminata*) was infected. In all cases that infections occurred, resistance was moderate, with a fraction of the available host cells becoming infected.

A group of 13 strains that did not belong to dinoflagellates, including representatives of diatoms, chlorophytes, haptophytes, raphidophytes, and cryptophytes, was also tested to determine the ability of the parasitoids to infect different phytoplankton phyla. None of the strains were infected (Supplementary Table 1).

**FIGURE 8:**
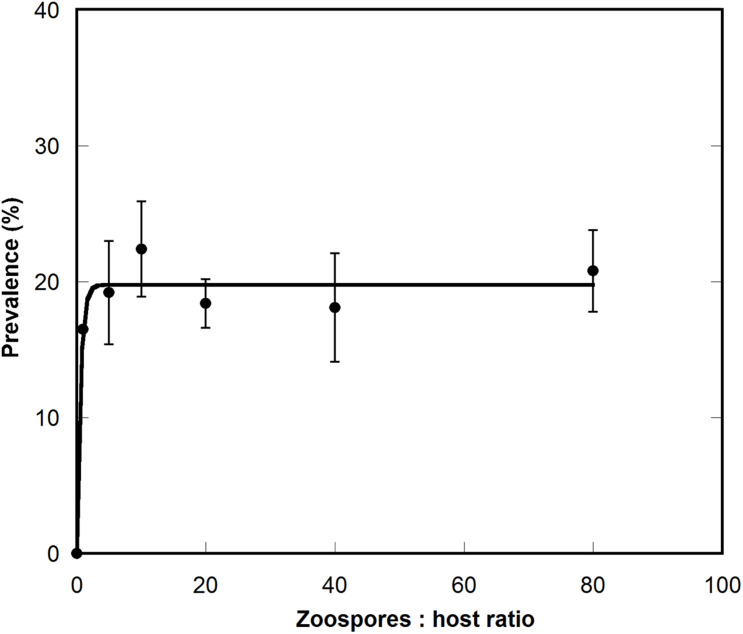
Prevalence curve of *Maranthos nigrum* infecting the dinoflagellate *Alexandrium minutum* Arenys strain. The X axis represents the ratio between zoospores and host cells, and the Y axis the percentage of infected cells. Whiskers represent the standard deviation between the three replicates.

Following qualitative observations showing generally low infection levels in the tested dinoflagellate species, prevalence curves were constructed to assess parasitoid abundance in *A. minutum*, as during the host-range experiments the infection rate of this dinoflagellate species was high. The results showed a maximum prevalence of 20%, reached at low zoospore:host cell ratios and maintained at higher ratios (Figure 8).

### Formal Description

Alveolata Cavalier-Smith, 1991

Myzozoa Cavalier-Smith and Chao, 2004

Perkinsea Levine, 1978

*Maranthos gen. nov.* Alacid et Reñé

DESCRIPTION: Endoparasitoid of dinoflagellates. Zoospores are biflagellated, with flagella equal in size and homomorphic. The two flagella emerge from the anterior half of the cell. After penetrating host cells, the sigmoid zoospores form an intracellular feeding stage (trophont). Following consumption of the host cytoplasm, the trophont enters a reproductive stage (sporont) that includes the formation of a smooth external cell wall. New zoospores are produced and, upon the collapse of this external sporangium wall, are subsequently released.

TYPE SPECIES: *Maranthos nigrum* sp. nov.

ETYMOLOGY: Marine flower, from mar- (marine) and ancient Greek –anthos (flower).

*Maranthos nigrum* sp. nov. Reñé et Alacid

DESCRIPTION: Endoparasitoid of dinoflagellates. The sporangium forms an external smooth wall whose collapse releases the zoospores. Zoospores possess bipartite trichocysts, a mitochondrion, and a refractile body. An apical complex is present, with a pseudo-conoid, micronemes and rhoptries. The zoospore nucleus includes dense heterochromatin along its periphery and a nucleolus.

HOLOTYPE: Figure 3E. An SEM stub containing the preserved free-living zoospores has been deposited at the Biological Reference Collections (CBR) of the Institut de Ciències del Mar (ICM-CSIC), Barcelona, under catalog number ICMCBR000465.

REFERENCE MATERIAL: An SEM stub containing preserved mature sporangia and three samples, embedded in resin for TEM, that were obtained from the type locality and contain all life-cycle stages of the parasitoid have been deposited in the Biological Reference Collections (CBR) (Guerrero et al., 2020) of the Institut de Ciències del Mar (ICM-CSIC) from Barcelona, under the catalog numbers ICMCBR000464, ICMCBR000466, ICMCBR000467, and ICMCBR000468, respectively.

TYPE LOCALITY: L’Estartit beach, Catalonia, NW Mediterranean Sea (42° 3′ 5″ N; 3° 12′ 9″ E).

TYPE HOST: *Alexandrium taylorii*

ETYMOLOGY: From Latin *nigrum*, referring to the characteristic black coloration of the central body as seen under optical microscopy.

## Discussion

### Phylogenetic Diversity of Perkinsea Infecting Dinoflagellates

The current diversity of Perkinsea is mostly represented by environmental sequences obtained in freshwater environments (Bråte et al., 2010; Mangot et al., 2011). Of the members that have been characterized thus far, all correspond to marine parasites, with the exception of the SPI agent infecting tadpoles (Chambouvet et al., 2015; Isidoro-Ayza et al., 2017). Among the identified parasitoids of dinoflagellates, all of the species have a close phylogenetic relationship, together forming the Parviluciferaceae family (Reñé et al., 2017a). In this study, the sequence obtained for Perkinsea *ex Barrufeta bravensis* was found to occupy a basal position in Parviluciferaceae, with moderate statistical support.

Additional environmental sequences cluster at the base of the Parviluciferaceae family with low statistical support. While all sequences of Parviluciferaceae species were obtained from marine environments, those environmental sequences correspond to freshwater environments (e.g., AY919729, AY919721 and AY919809 from a lake in United States, EU162625 and EU162627 from a lake in France) or from deep-sea sediments (e.g., HM103454 from Marmara Sea at 1260 m depth). Given the close relationship of the environmental sequences with Parviluciferaceae, some may correspond to parasites of dinoflagellates from freshwater environments.

*Maranthos nigrum* is also a parasitoid of dinoflagellates but its SSU rRNA gene sequence formed a long branch and there was no phylogenetic relationship with Parviluciferaceae or any other environmental sequence. This was not the case for the LSU rRNA gene sequences, which clustered at the base of Parviluciferaceae. However, the availability of only the LSU rRNA gene sequences of Parviluciferaceae and Perkinsidae prevented a thorough representation of the phylogenetic relationships for the currently known molecular diversity. The results for *M. nigrum* showed that parasitoids of dinoflagellates in Perkinsea are not restricted to those closely affiliated with Parviluciferaceae; rather, the diversity of dinoflagellate parasitoids may be much larger than currently considered. Since many available environmental sequences correspond to species from lakes and rivers (Jobard et al., 2020), and freshwater dinoflagellates are present in those environments, the existence of freshwater dinoflagellate parasites not affiliated with Parviluciferaceae should be explored.

### Comparison of the Morphological and Ultrastructural Features of *Maranthos nigrum*

A parasitic life style is common among many protist groups, such as Apicomplexa, Stramenopiles, Rhizaria, and Amoebozoa, all of which infect a wide range of hosts (Skovgaard, 2014). Some of those organisms, including the two species from this study, form motile infective zoospores during their life-cycle and are thus referred to as zoosporic parasites. These include epibiotic or ectoparasites such as chytrids or Ellobiopsea, which attach to the surface of host cells and then penetrate them to feed on the cytoplasm, as well as endobiotic or endoparasites such as Oomycetes, Rozellida, Blastocladiomycota, Syndiniales, and Perkinsea, which penetrate the host to develop within its cytoplasm (Gleason et al., 2014; Scholz et al., 2016). Among endobiotic parasites, some similarities can be found between those belonging to Syndiniales (marine alveolates) and *M. nigrum*. For example, *Euduboscquella cachoni*, a member of Syndiniales group I, mainly infects tintinnid ciliates (Coats, 1988) and its early trophonts are similar to those of *M. nigrum*. However, the mature trophonts are hemispherical, and the new sporocytes formed by successive divisions are arranged in chains. *Amoebophrya*, belonging to Syndiniales group II, also infects dinoflagellates, among other protist groups (Cachon and Cachon, 1987), but as in *E. cachoni* the trophont develops into a motile vermiform stage that dissociates into infective zoospores. Members of the genus *Syndinium*, belonging to Syndiniales group IV, infect copepods but also radiolarians (Skovgaard et al., 2005). In contrast to *M. nigrum*, they form a multicellular plasmodium inside the host that at maturity release new zoospores (Coats, 1999). Finally, *Coccidinium* species infect dinoflagellates and show a high similarity with Parviluciferaceae in general, and with *M. nigrum* in particular. However, the available information is mostly restricted to its formal description (Chatton and Biecheler, 1934, 1936), which has hindered a thorough determination of the features of *Coccidinium* despite its classification within Syndiniales. The life-cycle of *Coccidinium* is similar to that of the Parviluciferaceae, including the formation of a trophont that enlarges prior to mitosis, the presence of nuclei that accumulate in the cell periphery, and the production of new zoospores via gametogenesis (Chatton and Biecheler, 1934). The polymorphism of *Coccidinium*’s zoospores, with up to four different morphologies for a single species, was recognized early on (Chatton and Biecheler, 1936) but this has never been observed for Parviluciferaceae. The ability of *Coccidinium* spp. to maintain the host cell in a living state during trophont growth is also a feature of other Syndiniales, such as *Amoebophrya* (Chambouvet et al., 2011), whereas *M. nigrum* kills the host cell early in the infection stage. The differences suggest that *Coccidinium* and *M. nigrum* are not closely related, but this conclusion awaits further investigations of *Coccidinium*. Regardless, the morphological features of *M. nigrum* are distinct from those of other known parasites described in the literature but resemble those of Perkinsea members.

**FIGURE 9:**
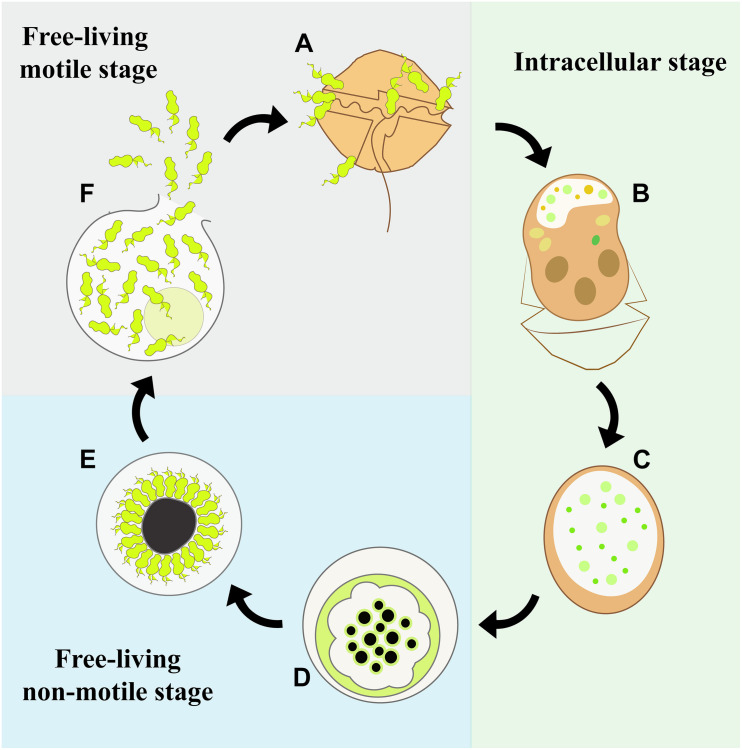
Diagram of the life-cycle stages of *Maranthos nigrum*. **(A)** A dinoflagellate cell is infected by free-living, motile zoospores. **(B)** Within this infected host cell, the parasitoid develops its life cycle starting from the feeding stage, when the host cytoplasm is consumed and an early trophont develops. The infected cell loses its theca and commonly acquires an elongated shape. **(C)** A late trophont has consumed all of the host cytoplasm. **(D)** It then develops into a free-living, non-motile sporont and replication to form new zoospores begins. **(E)** The zoospores develop inside the sporont, showing a radial disposition at the periphery of the cell outer membrane around a large central blackish body, which is distinctive of this species. **(F)** Once the zoospores are mature, the outer membrane ruptures and the zoospores are released into the environment.

The life cycle of *M. nigrum* is similar to that of other Parviluciferaceae species (Figure 9), in that the infection of a single dinoflagellate host is required for growth and reproduction, the completion of which is a prerequisite for further transmission. The parasite passes from one host to another via the free-living motile infective stage of its life cycle, the zoospore. Infection is followed by an intracellular stage, in which the parasitoid grows inside the host cell separated from the host cytoplasm by a parasitophorous vacuole membrane. The complete consumption of the host fosters the development of a reproductive free-living non-motile stage, the sporont, which subsequently gives rise to new zoospores, whose release into the environment and attachment to a host cell initiates the next round of infection. However, *M. nigrum* also differs remarkably from other members of Parviluciferaceae in terms of the morphology and ultrastructure of some of its life-cycle stages. For example, *Parvilucifera* species usually form one to two trophonts per host cell (Lepelletier et al., 2014; Reñé et al., 2017b), although three to five simultaneous infections are occasionally observed, especially during the infection of large dinoflagellate species (Garcés et al., 2013). By contrast, like *Dinovorax*, *Snorkelia*, and *Tuberlatum* which commonly develop multiple trophonts in the same host cell (Reñé et al., 2017a; Jeon and Park, 2019), in *M. nigrum*>3–5 trophonts were frequently seen within a single host cell. A unique feature of *M. nigrum* is that, during early infection stages, the developing trophont contains several small black bodies, as seen under light microscopy. In the sporonts of this species, the central body is large, granulated, and much darker than that of other known species. Another distinctive character of *M. nigrum* is the radial disposition of its zoospores around the large central body during the last stages of sporont maturation. The zoospores are similar in morphology and size to those of *Dinovorax*, including their sigmoid shape and the presence of two flagella of nearly the same size (Reñé et al., 2017a). By contrast, while the zoospores of *Tuberlatum coatsi* are also sigmoid, their flagella are heteromorphic (Jeon and Park, 2019) and the zoospores of other Parviluciferaceae representatives are reniform, elongated, or teardrop in shape, with flagella that are heteromorphic (Leander and Hoppenrath, 2008; Garcés and Hoppenrath, 2010; Lepelletier et al., 2014; Reñé et al., 2017b). Ultrastructurally, *M. nigrum* zoospores share features with those of *Dinovorax*, *Tuberlatum* and *Snorkelia* representatives, such as bipartite trichocysts (Leander and Hoppenrath, 2008; Reñé et al., 2017a; Jeon and Park, 2019), which are absent in *Parvilucifera* members. Also, unlike the processes-ornamented sporangium of *Parvilucifera* (Norén et al., 1999; Lepelletier et al., 2014; Reñé et al., 2017b), the external cell wall of *M. nigrum* is smooth, like that of *T. coatsi* (Jeon and Park, 2019), *D. pyriformis* (Reñé et al., 2017a), and *S. prorocentri* (Leander and Hoppenrath, 2008), but somewhat thinner.

The evolutionary strategy for zoospore release in Parviluciferaceae was not seen in either of the species described in this study. Among Parviluciferaceae members, the basal phylogenetic position is occupied by species that possess germ tubes, such as *Dinovorax* and *Snorkelia* (Reñé et al., 2017a), or short germ structures, such as *Tuberlatum* (Jeon and Park, 2019), while *Parvilucifera* representatives with a deeper position are characterized by circular opercula (Alacid et al., 2020). Perkinsea *ex Barrufeta bravensis* also occupies a basal position in Parviluciferaceae. Although its release structure could not be observed in detail, the absence of any type of germ tube to release the zoospores was clear. This finding contradicts previous suggestions that the morphology of zoospore-release structures allows an inference of the phylogenetic position within Parviluciferaceae. Among the other Perkinsea species, *Perkinsus* spp. zoosporangia also form a short discharge tube to release the zoospores (Sunila et al., 2001), but neither zoosporangia nor flagellated stages are seen in Xcellidae members (Freeman et al., 2017). For *M. nigrum*, in contrast to other species that infect dinoflagellates, its zoospores were not released through specialized structures, but after rupture of the external cell wall. This feature distinguishes *M. nigrum* from all other known Parviluciferaceae species.

### Ecology and Distribution of *Maranthos nigrum*

For parasites, life-history traits such as reproductive output (total offspring produced during an individual’s lifespan), mode of infection, and host range and specificity determine their transmission within host populations as well as their abundance and distribution in nature.

*Maranthos nigrum* was always detected in samples obtained during *A. taylorii* blooms from different geographical locations. *A. taylorii* produces seasonal blooms at many pocket beaches, covering a distribution area of around 100 km along the Catalan coast (Garcés et al., 1999). Recently, bloom events involving different dinoflagellate species were studied at several sites to determine the composition of the parasitic community and host-parasite interactions (Reñé et al., 2021). *M. nigrum* (as Perkinsea sp. 1) was detected only at beaches affected by *A. taylorii* blooms, in agreement with the results of our study. Clearly, *A. taylorii* is the preferred host of *M. nigrum*, at least along the Catalan coast, and the distribution of the parasitoid is linked to that of its dinoflagellate host. Similarly, Perkinsea ex *Barrufeta bravensis* (as Perkinsea sp. 2) was detected at beaches affected by *Gymnodinium litoralis* blooms (Reñé et al., 2021). *B. bravensis* and *G. litoralis* belong to the *Gymnodinium sensu stricto* clade and are thus phylogenetically closely related (Reñé et al., 2011, 2015; Sampedro et al., 2011). It can therefore be hypothesized that the preferred hosts of this newly identified parasitoid species are the naked dinoflagellates of this phylogenetic clade.

Interestingly, other parasite species belonging to Perkinsea were present during the *A. taylorii* and *G. litoralis* blooms (Reñé et al., 2021). The coexistence of parasites with similar life-cycles and host requirements can be explained by niche (host) partitioning and the different ecological strategies of the parasites. Although *M. nigrum* was able to infect several dinoflagellates, it may be less virulent than most Parviluciferaceae representatives, as during the host range experiment it infected fewer species than, e.g., *P. sinerae* (Garcés et al., 2013), *P. corolla* (Rodríguez and Figueroa, 2020), or *P. rostrata* (Lepelletier et al., 2014). Moreover, under laboratory conditions, its maximum prevalence in host populations was lower (∼20%) than that of other species, for which values >80% have been reported (Lepelletier et al., 2014; Alacid et al., 2016). This low virulence might be related to the life-history traits of *M. nigrum*, including the number of zoospores produced, swimming behavior, and host detection and infection. Like *D. pyriformis* (10–16 zoospores/10^3^ μm^3^ sporangium volume; (Reñé et al., 2017a) and *P. multicavata* (29 zoospores/10^3^ μm^3^; (Jeon and Park, 2020), *M. nigrum* produces fewer zoospores per sporangium than either *T. coatsi* (111 zoospores/10^3^ μm^3^) or *P. infectans* or *P. sinerae* (both 45 zoospores/10^3^ μm^3^ sporangium volume; (Jeon et al., 2018; Jeon and Park, 2019). Consequently, its zoospore:host ratio in the environment will not reach the high prevalence needed to ensure a high probability of host encounters and therefore successful infection and transmission (Park et al., 2004; Alacid et al., 2016). Moreover, the infection strategy of zoospores differs between different parasitoid species. While zoospores of *Parvilucifera* representatives are highly motile and have high rates of dispersion, with one or two zoospores often simultaneously penetrating a single host cell, zoospores of *M. nigrum* mostly form dense clouds that surround a few host cells (data not shown), leaving other cells free from parasitic infection. This infection strategy would explain both the low prevalence reached in the host population under laboratory conditions and the higher relative abundances of natural populations of *Tuberlatum* than of *M. nigrum* (as Perkinsea sp.1) during *A. taylorii* blooms (Reñé et al., 2021). The results of this study together with previous observations in nature suggest that different co-occurring parasitoids of dinoflagellates employ distinct ecological strategies to ensure their transmission and survival. A density-dependent strategy relies on strong peaks in parasite abundance during blooms of the respective host, whereas endemic infections ensure a low but consistent prevalence in natural host populations. As more Perkinsea parasitoids of dinoflagellates are described and become available in culture, detailed studies of their biological features and their swimming and infection behaviors will be possible. This knowledge will lead to a better understanding of the ecological strategies, abundance, and natural distribution of these organisms and ultimately their potential impact during coastal blooms of their dinoflagellate hosts.

## Data Availability Statement

The datasets presented in this study can be found in online repositories. The names of the repository/repositories and accession number(s) can be found in the article/Supplementary Material.

## Author Contributions

AR and EA designed the study. AR, EA, RG, and AF-V collected data. AC performed phylogenetic analyses. AR, EA, RG, AC, and EG drafted the manuscript. EG provided funds. All authors approved the final version of the manuscript.

## Conflict of Interest

The authors declare that the research was conducted in the absence of any commercial or financial relationships that could be construed as a potential conflict of interest.

## Publisher’s Note

All claims expressed in this article are solely those of the authors and do not necessarily represent those of their affiliated organizations, or those of the publisher, the editors and the reviewers. Any product that may be evaluated in this article, or claim that may be made by its manufacturer, is not guaranteed or endorsed by the publisher.

## Footnotes

1
http://iqtree.cibiv.univie.ac.at/

## Supplementary Material

Supplementary Figure 1 Maximum likelihood phylogenetic tree inferred from the LSU rRNA gene sequences of Perkinsea. Sequences of the Alveolata groups of Dinophyceae, Syndiniales I and Syndiniales II served as outgroups and sequences obtained in this study are indicated in bold. The bootstrap values (BS) and Bayesian posterior probabilities (BPP) are provided at each node (BS/BPP). Only BS and BPP values >70% and >0.95, respectively, are shown.

Supplementary Figure 2 Light microscopy micrographs showing some of the life-cycle stages of Perkinsea ex *Barrufeta bravensis.* **(A)** Healthy *Barrufeta bravensis* cell. **(B)** Early trophont (arrow) inside the host cell. **(C)** Double infection (arrows) on a collapsed host cell. **(D)** Early sporont. **(E)** Late sporont. **(F)** Empty sporangium. Arrowheads indicate the opercula. Scale bars = 10 μm.

Supplementary Figure 3 Light microscopy micrographs showing infections of *Maranthos nigrum* on different dinoflagellate species tested during the host-range experiment. **(A)** *Alexandrium affine*, **(B)** *A. minutum*, **(C)** *A. mediterraneum*, **(D)** *A. taylorii*, **(E)** *Coolia tropicalis*, and **(F)** *Scrippsiella trochoidea*. Arrows indicate parasitoid trophonts inside the host cell. Scale bars = 10 μm.

Supplementary Table 1 Strains of phytoplankton tested for susceptibility to infection by two strains of *Maranthos nigrum*. Yes/No indicate infection, and susceptibility indicates the infection capability on each host species, ranging from resistant: –; moderately resistant: +; moderately sensitive: ++; and sensitive: +++, following the classification from Lepelletier et al. (2014).
