# Supplementary figures and images for: New Perkinsea Parasitoids of Dinoflagellates Distantly Related to Parviluciferaceae Members
Source: Front Microbiol. 2021 Aug 5;12:701196. doi: 10.3389/fmicb.2021.701196 (PMC8375308; doi:10.3389/fmicb.2021.701196)

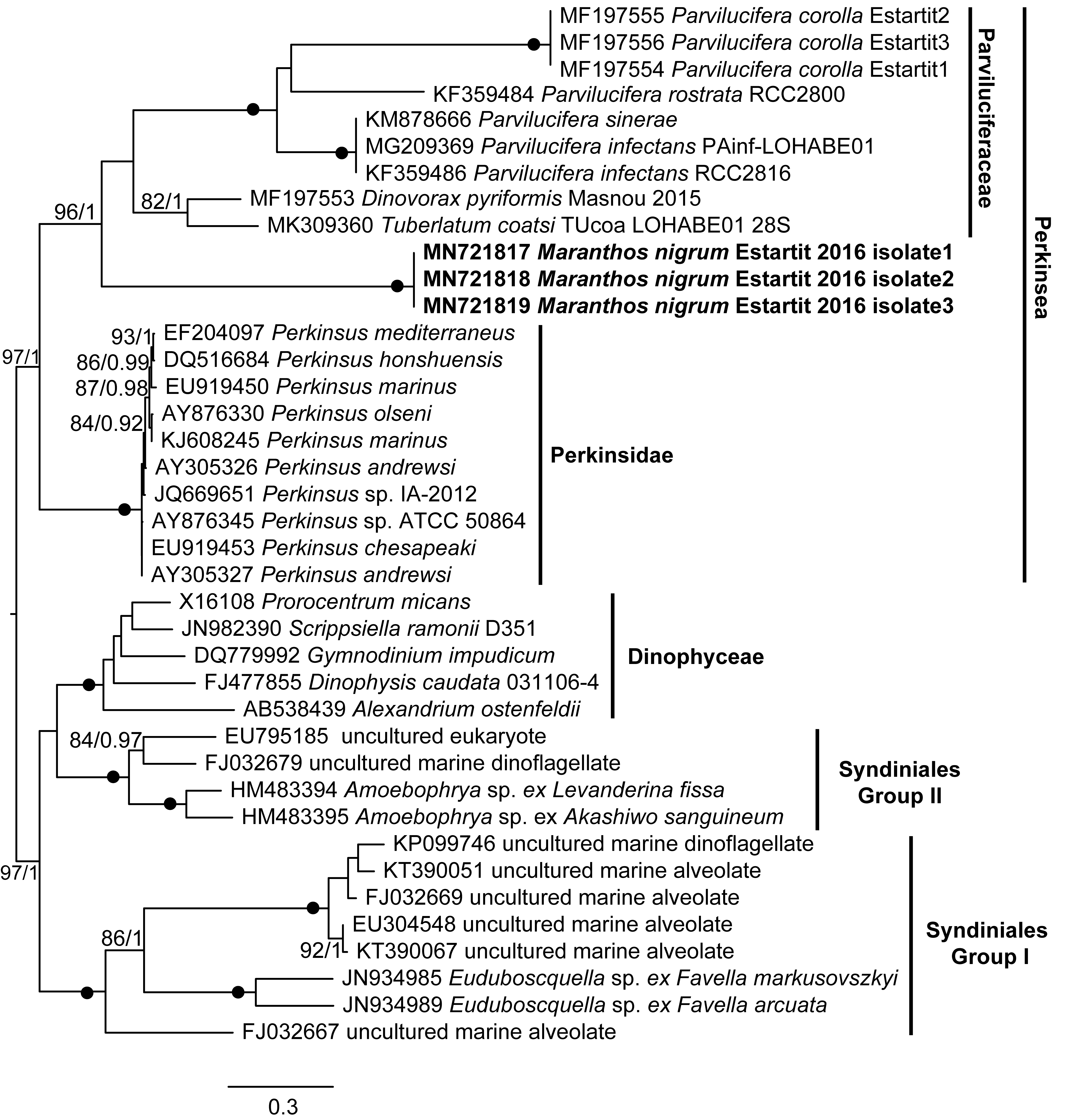

Supplement: Supplementary Figure 1 — Maximum likelihood phylogenetic tree inferred from the LSU rRNA gene sequences of Perkinsea. Sequences of the Alveolata groups of Dinophyceae, Syndiniales I and Syndiniales II served as outgroups and sequences obtained in this study are indicated in bold. The bootstrap values (BS) and Bayesian posterior probabilities (BPP) are provided at each node (BS/BPP). Only BS and BPP values >70% and >0.95, respectively, are shown. [file Image_1.tif]

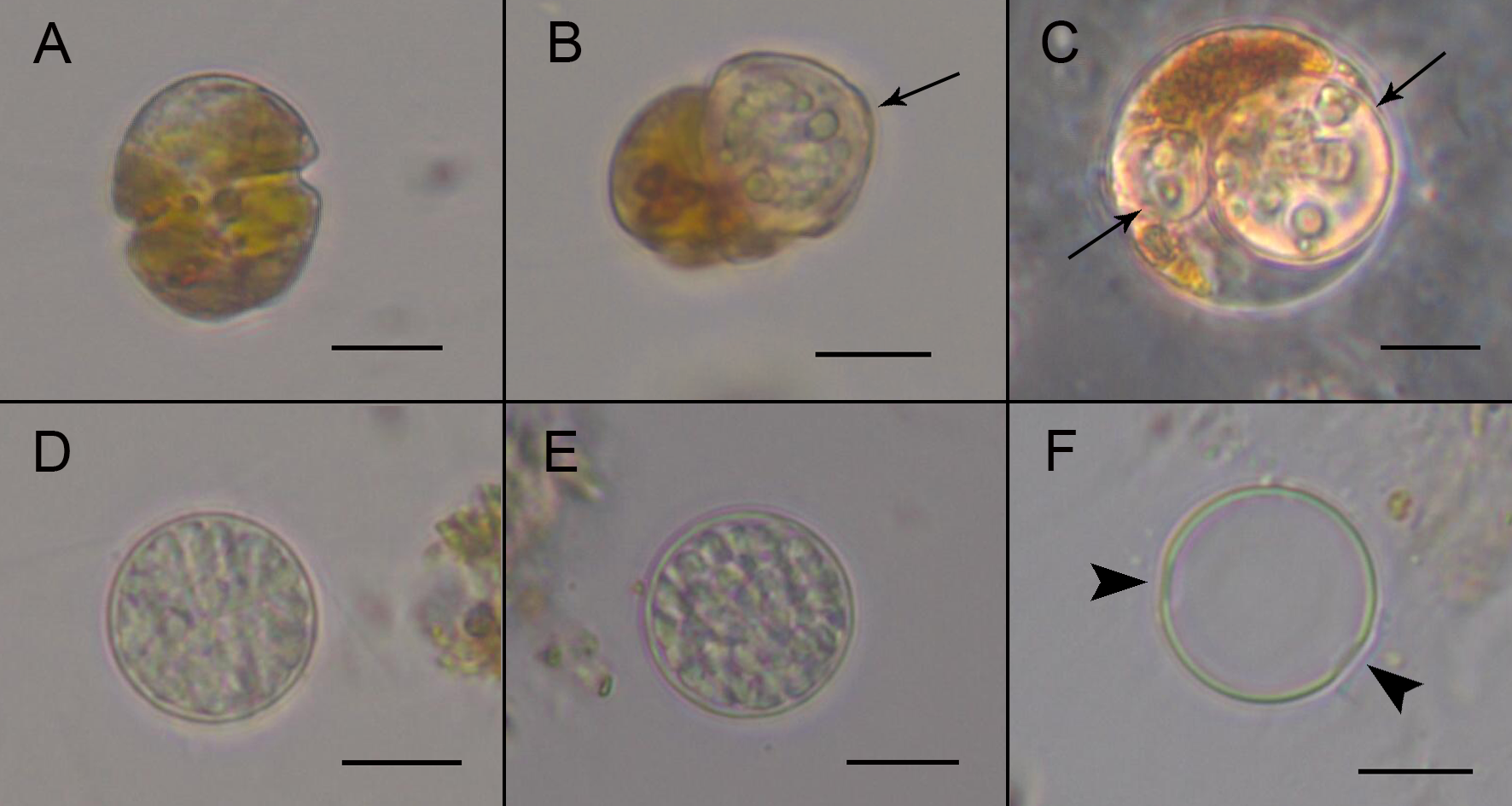

Supplement: Supplementary Figure 2 — Light microscopy micrographs showing some of the life-cycle stages of Perkinsea ex Barrufeta bravensis. (A) Healthy Barrufeta bravensis cell. (B) Early trophont (arrow) inside the host cell. (C) Double infection (arrows) on a collapsed host cell. (D) Early sporont. (E) Late sporont. (F) Empty sporangium. Arrowheads indicate the opercula. Scale bars = 10 μm. [file Image_2.TIF]

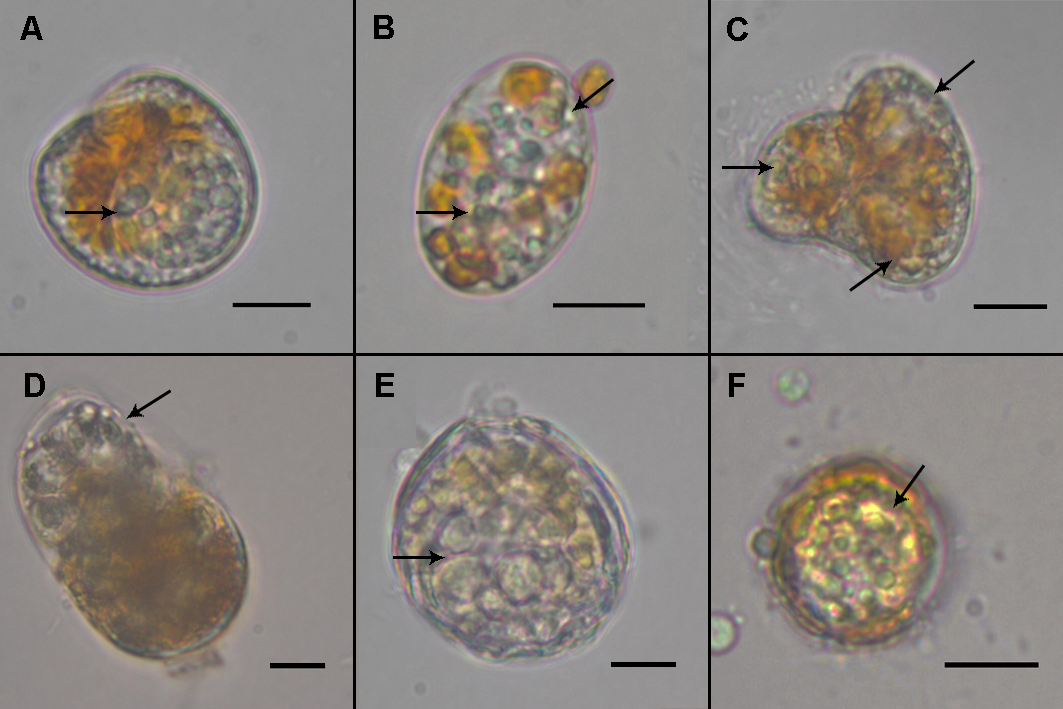

Supplement: Supplementary Figure 3 — Light microscopy micrographs showing infections of Maranthos nigrum on different dinoflagellate species tested during the host-range experiment. (A) Alexandrium affine, (B) A. minutum, (C) A. mediterraneum, (D) A. taylorii, (E) Coolia tropicalis, and (F) Scrippsiella trochoidea. Arrows indicate parasitoid trophonts inside the host cell. Scale bars = 10 μm. [file Image_3.TIF]
