# Supplementary material for: New Perkinsea Parasitoids of Dinoflagellates Distantly Related to Parviluciferaceae Members
Source: Front Microbiol. 2021 Aug 5;12:701196. doi: 10.3389/fmicb.2021.701196 (PMC8375308; doi:10.3389/fmicb.2021.701196)
Supplement: Supplementary Table 1 — Strains of phytoplankton tested for susceptibility to infection by two strains of Maranthos nigrum. Yes/No indicate infection, and susceptibility indicates the infection capability on each host species, ranging from resistant: –; moderately resistant: +; moderately sensitive: ++; and sensitive: +++, following the classification from Lepelletier et al. (2014). [file Table_1.DOCX]

Supplementary Material

**Supplementary Table 1: Strains of phytoplankton tested for susceptibility to infection by two strains of *Maranthos nigrum*.** Yes/No indicate infection, and susceptibility indicates the infection capability on each host species, ranging from resistant: -; moderately resistant: +; moderately sensitive: ++; and sensitive: +++; following the classification from Lepelletier et al. 2014.

| **Phylum/Class** | **Order** | **Genus** | **Species** | **Strain** | ***M. nigrum* strain** | | **Susceptibility** |
| --- | --- | --- | --- | --- | --- | --- | --- |
|  |  |  |  |  | **Estartit**  **2017** | **Sant Pol 2018** |  |
| Dinoflagellate | Dinophysales | *Dinophysis* | *acuminata* | VGO1391 | No | No | - |
|  | Gonyaulacales | *Alexandrium* | *affine* | PA8V | Yes | Yes | + |
|  |  |  | *andersonii* | CCMP2222 | Yes | Yes | + |
|  |  |  | *catenella* | Chile | Yes | Yes | + |
|  |  |  | *margalefi* | VGO661 | No | No | - |
|  |  |  | *mediterraneum* | BT30 | Yes | Yes | + |
|  |  |  | *minutum* | AMP4 | Yes | Yes | + |
|  |  |  | *minutum* | Arenys | Yes | Yes | + |
|  |  |  | *minutum* | VGO657 | Yes | Yes | + |
|  |  |  | *tamarense* | SZN029 | Yes | Yes | + |
|  |  |  | *taylorii* | - | Yes | Yes | + |
|  |  | *Gambierdiscus* | *excentricus* | VGO1198 | No | Yes | + |
|  |  | *Coolia* | *monotis* | VGO831 | No | No | - |
|  |  | *Coolia* | *tropicalis* | VGO923 | Yes | Yes | + |
|  |  | *Ostreopsis* | *ovata* | OOPM17 | Yes | Yes | + |
|  | Gymnodiniales | *Amphidinium* | *carterae* | AO1BR | No | No | - |
|  |  | *Barrufeta* | *bravensis* | VGO866 | No | No | - |
|  |  | *Gymnodinium* | *catenatum* | VGO744 | No | No | - |
|  |  | *Gymnodinium* | *impudicum* | GY4VA | No | No | - |
|  |  | *Gymnodinium* | *litoralis* | B12 | No | No | - |
|  |  | *Karlodinium* | *veneficum* | K21 | No | No | - |
|  |  | *Levanderina* | *fissa* | - | No | No | - |
|  | Peridiniales | *Heterocapsa* | *triquetra* | RCC4813 | Yes | Yes | + |
|  |  | *Kryptoperidinium* | *foliaceum* | BAIONA | Yes | Yes | + |
|  |  | *Scrippsiella* | *trochoidea* | S3V | Yes | Yes | + |
|  | Prorocentrales | *Prorocentrum* | *hoffmanianum* | 230315 | No | No | - |
|  |  |  | *micans* | PM1V | No | No | - |
|  |  |  | *minimum* | AND1V | No | No | - |
|  |  |  | *rathymum* | VGO680 | Yes | Yes | + |
|  |  |  | *triestinum* | PT3V | No | No | - |
|  |  |  | *lima* | PL20V | Yes | Yes | + |
| Diatom | Coscinodiscales | *Coscinodiscus* | *radiatus* | CCMP312 | No | No | - |
|  | Thalassiosirales | *Thalassiosira* | *weissflogii* | CCAP1085 | No | No | - |
|  | Chaetocerotales | *Chaetoceros* | *affinis* | LA30 | No | No | - |
| Chlorophyta | Pyramimonadales | *Pyramimonas* | sp. | PY01V | No | No | - |
|  | Chlorodendrales | *Tetraselmis* | *convulatae* | VGO | No | No | - |
|  |  |  | *suecica* | CCAP66 | No | No | - |
| Haptophyta | Coccolithales | *Holococcolithophora* | *sphaeroidea* | TGN-ICM | No | No | - |
|  |  | *Pleurochrysis* | *elongata* | CCMP874 | No | No | - |
|  | Isochrysidales | *Emiliania* | *huxleyi* | EH02V | No | No | - |
|  | Prymnesiales | *Prymnesium* | *faveolatum* | VGO557 | No | No | - |
| Raphidophyceae | Chattonellales | *Chattonella* | *subsalsa* | VGO1207 | No | No | - |
|  |  | *Heterosigma* | *akashiwo* | HA1V | No | No | - |
| Cryptophyceae | Pyrenomonadales | *Rhodomonas* | *baltica* | CCAP979 | No | No | - |
